# Political economy analysis of health financing reforms in times of crisis: findings from three case studies in south-east Asia

**DOI:** 10.1186/s12939-025-02395-5

**Published:** 2025-02-03

**Authors:** Sophie Witter, Maria Paola Bertone, Sushil Baral, Ghanshyam Gautam, Saugat K. C. Pratap, Aungsumalee Pholpark, Nurmala Selly Saputri, Arif Budi Darmawan, Nina Toyamah, Rizki Fillaili, Valeria de Oliveira Cruz, Susan Sparkes

**Affiliations:** 1 Institute for Global Health and Development, Queen Margaret University, Musselburgh, Edinburgh, EH216UU UK; 2 ReBUILD for Resilience Research Consortium, Liverpool, United Kingdom; 3 HERD International, Kathmandu, Nepal; 4 Faculty of Social Sciences and Humanities, Mahidol University, Nakhon Pathom, Thailand; 5 SMERU Research Institute, Jakarta, Indonesia; 6 World Health Organisation, Southeast Asia office, Delhi, India; 7 World Health Organisation, Geneva, Switzerland

**Keywords:** Health financing, Reforms, Political economy analysis, Shocks or crises, Nepal, Thailand, Indonesia

## Abstract

**Background:**

Over the last decades, universal health coverage (UHC) has been promoted in south-east Asia (SEA), where many countries still need to ensure adequate financial protection to their populations. However, successful health financing reforms involve complex interactions among a range of stakeholders, as well as with context factors, including shocks and crises of different nature. In this article, we examine recent health financing reforms in Nepal, Thailand and Indonesia, using a political economy lens. The objective is to understand whether and how crises can be utilised to progress UHC and to analyse the strategies used by reformers to benefit from potential windows of opportunity.

**Methods:**

The study adopted a retrospective, comparative case study design, using a shared framework and tools. The case studies mapped the contexts, including economic, political, social trends and any shocks which had recently occurred. A focal health financing reform was chosen in each setting to examine, probing the role of crisis in relation to it, through the key elements of the reform process, content and actors. Data sources were largely qualitative and included literature and document review (144 documents included across the three cases) and key informant interviews (26 in total).

**Results:**

The findings, which bring out similarities and differences in the roles played by change teams across the settings, highlight the importance of working closely with political leaders and using a wide range of strategies to build coalitions and engage or block opponents. Changing decision rules to block veto points was significant in one case, and all three cases used participation and dialogue strategically to further reforms. More broadly, the links with context emerged as important, with prior conflicts and economic crises creating a sense of urgency about addressing health inequities, while in all countries appeal was made to underlying values to enhance the legitimacy of the reforms.

**Conclusion:**

The lessons from these case studies include that technical teams can and should engage in Political Economy Analysis (PEA) thinking and strategizing, including being aware of and adaptable to the changing PEA landscape and prepared to take advantage of windows of opportunity, including, but not limited to, those emerging from crisis. There is a need for more empirical studies in this area and sharing of lessons to support future reforms to increase health coverage and financial protection, including in the face of likely shocks.

**Supplementary Information:**

The online version contains supplementary material available at 10.1186/s12939-025-02395-5.

## Background

Over the past two decades, many governments, including in the SEA region, have sought to promote equitable access to quality health services by reforming their health financing policies and expanding effective coverage policies. However, broad agreement on the importance of UHC has not translated easily into the successful implementation of related health financing reforms and as a result the region lags behind when it comes to both UHC measures: service coverage index stood at 62 (out of 100) against the global average of 68 and lack of financial protection^1^ reached 16 while the global average was 13.5% in 2019 [1]. Health financing reform that focuses on expanding effective coverage involves complex interactions among a range of stakeholders in the health sector and beyond, with varying power and influence. Added to this are dynamic economic factors that directly impact feasible reform options. This - and the redistribution that is inherent in many health financing reforms that focus on UHC expansion - can make reform politically contentious and difficult to move forward to adoption and implementation [2]. By understanding the various stakeholders and institutions involved in health financing reform, their interests, relative power, along with related economic factors, strategies can be developed to overcome or take into account their positions. This involves identifying, and being ready to take advantage of, windows of opportunity when they emerge. The objective of incorporating political economy analysis in this way is to support a more strategic and proactive approach to reform as a way to increase the likelihood of implementation and ultimately progress towards UHC [3].

Historically, crises of various kinds have enabled certain countries to enact policies that dramatically expand coverage and ultimately improve the health of their populations [4]. In those cases, crises have provided an identifiable problem that has raised public awareness, but as theory tells us [5], the precise policies and politics will ultimately need to come together to move reform forward. In order to progress towards UHC, there is a need to understand the political and economic dynamics, along with other factors, that facilitated change in countries emerging from previous crises, and the various strategies pursued to move forward reforms. This is particularly relevant in the SEA region, where many countries’ health financing system still fail to ensure adequate financial protection to large shares of their populations [6].

In this article we examine health financing reforms to progress UHC in three countries in SEA (Nepal, Thailand and Indonesia) over the past two decades, using a political economy lens and focusing on understanding the role of crises, and how these enabled or modified reforms. The objective is to understand whether and how crises can be utilised to progress UHC and to analyse the strategies used by reformers to benefit from the window of opportunity that they may open. There is limited empirical investigation of this area to date; studies have investigated typical UHC reform trajectories and the politics of UHC, but not with a strong focus on crisis, nor with a clear focus on stakeholder management strategies [7, 8]. Analysis of these can bring out lessons for future reformers.

## Methods

### Study design

The study adopted a retrospective, comparative (rapid) case study design. Three countries were selected purposively to include countries in the region which had recently undertaken major health financing reforms with a range of outcomes (some recognised as successfully, such as Thailand’s, others with partial success, such as Nepal’s) (Box 1). A focal health financing reform was chosen in each setting – based on its salience in health financing reforms of the past two decades in each country - to examine in more depth, probing the role of crisis in relation to it, through the key elements of the reform process, content and actors. Initially, the three cases were developed separately [9–11]. However, the framework and tools adopted were the same for all studies, which allowed us at a second stage to compare and contrast findings, reflect on patterns and similar or divergent features, and strengthen the generalisability of the findings and of the lessons learned.

The paper, and embedded cases, use applied political economy analysis (PEA) based on the World Health Organization’s “Political economy analysis for health financing: a how to guide,” which lays out a structured way to analyse political economy dynamics that impact health financing reform processes and related outcomes [3]. This guide builds from the approach laid out by Reich and Campos (2019) focusing on both the role of “change teams” (i.e. people taking a role in promoting reforms that are needed to improve the health financing functions in their province, region or country) and the relevant stakeholder groups that have an interest, position and power with respect to the reform [12]. The framework, with the WHO guide, adds considerations around the contextual factors that impact reform trajectories and outcomes. In this way, the PEA approach used in this study involves a review of the contextual (structural) factors unique to each country and a careful assessment of relevant stakeholders, their power, position and interest in the health financing reform, as well as of the formal and informal institutions through which they interact [3].

PEA is not based on a single theory, but has as a central contention that public goods arise through political processes and contestation between interest groups, enacted through the interaction of formal and informal institutions. Political settlements or bargains can be reached amongst elites, which can be stable but can also potentially be shifted by acute events which challenge the status quo and create a window of opportunity for reform [13, 14]. One framework for thinking about such windows was developed by Kingdon, who hypothesised that for reforms to occur successfully, three ‘streams’ needed to converge: the problem stream (perceptions of the urgency of the problem), the policy stream (availability of policies to address the problem), and the politics stream (a supportive constellation of actors) [5]. In keeping with that, the case studies started by mapping the contexts, including economic, political, social trends and any shocks which had occurred over the two decades.

The cases directly demonstrate the politics stream of Kingdon’s multiple stream framework [5]. The cases were selected based on the existence of the policy and problem streams and the PEA then assessed how the politics stream did or did not come together to determine outcomes with the other three streams, and how this was affected by crisis. The discussion considers elements of all three streams in determining reform outcomes.

### Study settings

Until COVID-19 hit, SEA region was the fastest growing region in the world. The annual per capita Gross Domestic Product (GDP) growth rate for the region on average was 4% from 2009 to 2019 – with countries annual estimated GDP growth rates in Bangladesh, India, and Myanmar exceeding 5% per year. However, the region still has one of the highest poverty rates in the world. When compared to other World Health Organization (WHO) regions, SEA has the smallest health sector (as measured by current health spending as percent of GDP), and most countries spent less on health than countries with similar income levels in other regions. Domestic government spending on health continued to be low, comprising less than 50% of the entire health spending in five out of 10 countries in the region. As a result, SEA remains the region with the highest share of out-of-pocket (OOP) health spending at 36% in 2021 [15]. The case study countries within the region were selected to represent a range of contexts and degrees of success in introducing UHC reforms.

#### Thailand

Thailand stands out in the region, as one of the countries that has made remarkable progress towards UHC, with a growth in GDP per capita, reduction in poverty rates, substantial increases in current health expenditure (CHE) per capita, and a reduction in reliance on out-of-pocket (OOP) spending from 34% of CHE to 9% over 2000–2023 (Table 1). The share of the population affected by household catastrophic health expenditure stood at 2% of population in 2017. In the same year, 0.01% were pushed into poverty because of OOP health expenditures, down from 0.06% in 2009 (under the daily US$3.20 per capita poverty line). The UHC health financing reform of 2002 described below has been instrumental in supporting the country in containing health care costs and improving strategic purchasing and accountability for public resources, while ensuring wide coverage and in-depth financial protection [16].

**Table 1:** Overview of key context variables

Variable	Period	
**Nepal**	**1990s**	**Latest Year**
Population size (million)	18.5 (1991)	29.93 (2022)
GDP/capita	194.8 (1991)	1,348.2 (2022)
Poverty incidence (%)	42 (1996)	20.3 (2022)
CHE per capita, USD	8 (2000)	65 (2021)
OOP as % CHE	55.8(2000)	51.3 (2021)
**Thailand**	**1990s**	**Latest year**
Population size (million)	61.88 (2000)	66.05 (2023)
GDP/capita (current USD)	1,841 (1998)	7,171.8 (2023)
Poverty incidence (%)	38.63 (1998)	5.43 (2022)
CHE per capita, USD	62 (2000)	364 (2021)
OOP as % CHE	34 (2000)	9 (2021)
**Indonesia**	**1997/8**	**Latest year**
Population size (million)	204.6 (1997)	277.50 (2023)
GDP per capita (current US$)	459 (1998)	4,940 (2023)
Poverty incidence (%)	27 (1998)	9.4 (2023)
OOP as % CHE	45.2% (2000)	27.5% (2022)

Sources: Indonesia population size [81], GDP per capita [82], poverty incidence [61, 83], OOP [84]; Thailand population size [85], GDP per capita [86], poverty incidence [87], CHE per capita & OOP [15]

#### Nepal

While Nepal has made progress towards UHC, its successes are not yet at the level of Thailand, with more modest growth in GDP and dependence on OOP expenditure dropping from just under 56% in 2000 to just over 51% in 2021 (Table 1). In 2014, the proportion of the population affected by household catastrophic health expenditure stood at 11%. In the same year, about 2% of the population were impoverished due to OOP health payments (under the daily US$1.90 per capita poverty line). A series of recent reforms in Nepal, described below, have been introduced in Nepal to address these issues and make progress towards UHC.

#### Indonesia

The progress towards UHC in Indonesia has been positive over the past decade, though less dramatic than Thailand’s. With growth, reductions in poverty and reforms came a drop in reliance on OOP spending in health, halving from 45% in 2000 to 27.5% in 2022 (Table 1). In 2021, the proportion of the population experiencing household catastrophic health expenditure decreased to 2%, down from over 3% in 2013 [17]. Meanwhile, the percentage of the population impoverished due to OOP health payments was 0.7% in 2018, based on the daily US$3.20 per capita poverty line [18]. The introduction of SHI in 2014 significantly improved access to healthcare by eliminating the previously fragmented health insurance schemes. Currently, over 95% of Indonesia’s population is registered with SHI.

### Data sources

Data sources were largely qualitative and included an extensive literature and document review and a series of key informant interviews.

In each country, the document review focused on published documentation such as peer-reviewed articles, grey literature and reports, policies, strategies, and laws referring to the health financing reforms in the country during the period from the year 2000 up to 2021. Further information was collected on key socio-economic, political and epidemiological events, including important turning points (elections, constitutional reviews, etc.) and crises and shocks.

A series of Key Informant Interviews (KIIs) was carried out with the purpose of filling in specific gaps in information identified by the documentary review and gathering further information, in particular reflecting the views and perceptions of key actors involved in the reform processes. The KIIs helped to illuminate the links between the crisis/shock and the health financing reforms – for example, in terms of how specific strategies to support reform processes were defined and adapted to reflect the context and the features of the crisis. KIIs also focused on the positioning of actors, the PEA strategies adopted and reflections on their impact.

### Documentary data

Documents on health financing reforms were collected through a purposeful, non-systematic search with an iterative approach. Initially, the search was carried out in on search engine and databases (specifically, Google Scholar (first 100 hits) and PubMed). The search included key words such as “health financing”, “health financing reforms”, “Universal Health Coverage”, alone as well as in conjunction with “political economy” “political economy analysis” “health policy”, “policy processes”, and was carried out with reference to the specific country cases. Publications since 1997 were screened (this starting point relates to the Asian Financial Crash, which was influential for both the Thai and Indonesian reforms). Secondly, targeted searches were run in institutional websites (i.e., respective Ministries of Health, WHO, World Bank). In addition, we reviewed references of each identified document and asked key informants to share any additional relevant documents. A second, targeted search was conducted in order to map the context, using pre-identified categories of information drawing from existing tools [3]. The search aimed to identify documents addressing questions around the macro-economic and fiscal, historical, political, institutional, cultural and global context. Because our analysis took a long-term perspective covering two decades, key shifts (and the nature and timing of those changes) in the context being mapped have been described.

For the Nepal case study, 50 documents were selected. The majority [31] of the documents looked at health insurance in Nepal and the remaining looked at the maternity incentive scheme, macroeconomics and health in Nepal, and examining OOP expenditures.

For Thailand, 49 documents were selected as relevant. 26 published articles or reports referred directly to the Universal Coverage Scheme (UCS) reform of 2002 (our case study) as single case or in a comparison of case studies and adopted a specific policy analysis or PEA perspective. Other documents looked at related topics, for example detailing the technical content of the reform and assessing its impact(s) and performance, or expanding the reflection, focusing on research capacity, role of evidence and knowledge management for health financing reforms, and participatory bodies (such as the National Health Assembly).

For Indonesia, 45 documents were selected. Sixteen articles discussed UHC and SHI in Indonesia as a single case or in comparison with health insurance schemes in other countries. Other documents focused on the decentralization process and progress, the impact of financial crisis on the health reform, and PEA of health financing from other countries.

### Key informant interviews

Our sample of key informants included 26 participants – 12 in Nepal, 8 in Indonesia and 6 in Thailand (Table 2). Participants were identified based on the literature, suggestions from the WHO regional and country offices, knowledge of the research team, or suggestions from informants themselves during interviews. Participants were high-level individuals with a good historical overview of the reform processes in the health financing sub-sector and included representatives of Ministry of Health (MOH) and other governmental bodies, current and former politicians, academic, research institutions and think tanks, donors and UN agencies, and other key constituencies as relevant in each country. Sampling was purposeful, starting from the literature but augmented by suggestions from the KI themselves as well as the research team and WHO offices, with the aim of being representative of actors involved in health financing reforms, having recall of the relevant events, being available and willing to talk. In some cases, representatives of specific categories were omitted due to lower relevance to the case study (e.g. external actors in Thailand) or due to challenges gaining their participation (e.g. political leaders in Indonesia). Respondents were contacted via email to explain the purpose of the study and invite their participation. Those who agreed to participate were asked to read and sign an information sheet and consent form. Interviews were conducted using a semi-structured topic guide (supplementary file A) by the research team in person and remotely, either in English or in local languages. They lasted 45 min to one hour. They were recorded with the consent of the participants and notes taken during the interviews.

**Table 2:** Summary of key informant interviews

Respondent group	Nepal	Thailand	Indonesia
Bureaucracy (e.g. Ministries of Health staff, executive agencies)	2	2	4
Political leaders (e.g. senior leadership, retired political office holders)	2	2	
External actors (e.g. development partners in health sector)	4		1
Beneficiaries (e.g. civil society, NGOs)	1	1	1
Technical experts/researchers/academics	3	1	2
Total	12	6	8

### Data analysis and synthesis

An initial descriptive analysis of the data was carried out by extracting data from documents and coding interviews, using a thematic content analysis approach, which is appropriate where there is an initial set of questions and an interest in comparing findings across cases [19]. Coding was done manually using an MS Excel template by the researcher team. An extraction matrix and a coding tree were developed for this purpose, mostly based on pre-identified themes but also with additions of emerging codes and themes (deductive/inductive approach). Themes and codes reflect the political economy approach of the study, focusing on the agency (actors) and structural (context) elements that influence policy-making dynamics, as well as processes and policy content (also highlighted in the structure of the topic guide) (Supplementary file A).

Based on coded data, we have analysed the content of the reform or series of reforms in each setting, as well as the context of the reforms, focusing on the socio-economic, epidemiological and political events in each country and with particular attention to the specific shock or crises for each country, and their influence on health financing reform processes.

Secondly, a stakeholder mapping was developed (focusing on a specific point in time) in order to clearly map the level of power and interest of key stakeholders [20]. This allowed unpacking of the relations and power dynamics between actors, how those might have been influenced by crisis and how they affected the health financing reform processes. For the mapping, the categories proposed by Campos and Reich (2019) were adopted [12]. They group stakeholders into 6 + 1 categories: [1] interest groups [2], bureaucracy [3], budget-related groups [4], leadership [5], beneficiaries, and [6] external actors. All these relate with the last (central) category of health sector policymakers (“change teams”).

Finally, analysis was carried out to identify specific strategies (including negotiation, coalition building, compromises, sequencing of reforms) adopted by key stakeholders to ensure the progress of health financing reforms [20]. Findings have been integrated and synthetized using the framework proposed by Campos and Reich (2019).

### Ethical approval

Ethical approval was obtained from the Nepal Health Research Council (for the Nepal case study), from Commission of Ethics in the Social Humanities Research, National Research and Innovation Agency (for the Indonesia case study), and from Mahidol University (for the Thailand case study).

## Results

We first describe the set of reforms on which our case studies focus. In the case of Thailand, we focus on the 2002 UCS reform moment, while in Nepal the series of reforms bringing in conditional cash transfers, waivers and exemptions for maternal health services and then waivers and exemptions for basic health care over 2005–2020 is our focus. In Indonesia, it is the flagship *Jaminan Kesehatan Nasional* (JKN/SHI) reforms since 2014. Next, we briefly describe the context of health financing reforms within each of the case studies. We then focus on the stakeholder roles and analyzed their strategies, and how these influenced (and were influenced by) the reform design, adoption, implementation and results, as well as by crises.

### Content of health financing reforms

This section outlines the content of the focal health financing reforms. Further details on the process of their development, their drivers and content can be found in the country studies [9–11].

#### Nepal

In 2005, the Maternity Incentives Scheme was launched – a demand side finance approach, which was supplemented by free maternity services in 2006, when the policy was relabelled the Safe Delivery Incentive Programme (SDIP). From this point, a dynamic of adding ‘free care’ components was established. The programme later developed into the Aama programme, in 2008/9, with payments for deliveries made to facilities (to cover services costs), staff (for incentives) and households (to cover access costs), tiered by ecological region. In October 2007, the Government of Nepal declared all health services at health posts and sub-health posts free of charge to all, so people could more easily access and use basic health care services [21]. In January 2009, universal free care was extended to primary healthcare centres, and free outpatient care for targeted groups was expanded to all districts. This included the provision of 40 essential drugs free of charge at district hospitals, and deliveries became free for all women at government institutions nationwide [22]. These policies aimed to reduce financial barriers to seeking delivery care, provide relief to poor families, promote the utilization of essential health care services, increase maternal and newborn survival, and ultimately improve the health status of women and newborns. Free newborn care was added to the package in 2016 in recognition of the effectiveness and popularity of the Aama programme.

The 2015 constitution of Nepal declared basic healthcare as a fundamental right of citizens, placing the responsibility of financing such care on the central state and the delivery of basic health services on local governments. Additionally, increasing state investment in the health sector and providing health insurance for all were defined as state policies in the 2015 constitution. A specific package of basic health services was defined through the enactment of Public Health Service Act in 2018 and its Regulations in 2020. The list of free essential drugs has been widened to 98 types, which are being supplied primarily through basic health care facilities. Additionally, in 2016, the government introduced a health insurance scheme to cover services beyond the basic health services package. A national health insurance bill was passed in 2017, which enabled expansion of the pre-established health insurance scheme, aiming to ensure quality healthcare through the prepayment and pooling of financial resources. The state covers premium for the citizens identified as poor and target categories, and further complements with substantial budgetary support, making it a tax-financed scheme in essence. More recent reforms include the provision of free essential drugs and free treatment for patients of specific disease conditions, which include cardiovascular disease, cancer, spinal injuries, renal ailments, and sickle cell anemia through the Disadvantaged Citizens Medical Treatment Fund [23].

These reforms have contributed to widening access to health services, but challenges remain in fully implementing these policies. The initiation of these various schemes reflects the government’s commitment to improving public health and reducing inequalities. However, the political commitment has not been backed up by the required resources, thus leading to weak results. For example, the percentage of facilities having the tracer medicines necessary to provide quality care was found to be 41% in 2021 [24]. Similarly, despite the expansion of the publicly administered health insurance scheme throughout the country, only 16% of the population maintained active enrollment status as of the fiscal year 2022/23 and nearly one-quarter of the people did not renew their enrollment status [25]. The UHC service coverage index is still low at 53 and OOP expenditure remains the dominant source of financing [24]. Moreover, the multitude of schemes has led to fragmentation in the health financing architecture. Such challenges were the drivers for the development and endorsement of a national health financing strategy in 2023, which has been on the agenda for more than a decade. This recent strategy focuses on mobilizing equitable resources for health towards achieving UHC [26].

#### Thailand

During the period between the 1970s and 1990s, Thailand had made progress towards establishing prepayment mechanisms for different groups within its population. These included the Medical Welfare Scheme (MWS) covering the poor, elderly, disabled, children under 12 years old and other vulnerable population groups, the Civil Servant Medical Benefit Scheme (CSMBS) for government employees, including their dependents, the Social Security Scheme (SSS) for private-sector employees, and the Voluntary Health Card Scheme, as a voluntary scheme for self-employed and/or informal workers. However, by the early 2000s, 29% of the population was still not covered by any form of health insurance [27, 28].

In order to extend coverage to the entire population and achieve UHC, the 2002 National Health Security Act envisaged the introduction of the UCS. The UCS was a tax-funded scheme, initially with a co-payment of 30 Baht per visit or admission (which was then terminated in November 2006). It was established by merging two existing schemes, the MWS and the Voluntary Health Card Scheme, with increased funding from the government (30 billion Baht) which allowed to cover the 30% uninsured population. It did not substitute the existing CSMBS and SSS schemes, but complemented those two by covering the population groups that did not have access to them [28].

The UCS established a comprehensive benefits package with a primary care focus and gatekeeping function [28]. The initial benefit package was designed based on existing ones and in particular the MWS one, and included outpatient, inpatient, medicine and other high-cost services. Subsequent revisions of the benefit package were mandated to be guided by health technology assessment, including cost-effectiveness analysis, budget impact assessment, equity and ethical considerations, and supply-side capacity to scale up. Major additions over time were Anti-Retroviral Therapy (ART) in 2006 and renal replacement therapy in 2008 [29].

Strategic purchasing was introduced with a fixed annual budget per member (capitation based) and a cap on provider payments (age adjusted capitation for outpatient services, and Diagnostic Related Groups (DRG) within an annual global budget for inpatient services), which aimed to put a “hard” limit to the budget and contain costs. Overtime, the capitation payment increased from 1202 Baht per capita in 2002 and to 2693.5 Baht in 2011 due to expansion of the benefit package, labour costs and medical products inflation [27, 28].

The reform also introduced a radical reorganisation of the Ministry of Public Health (MOPH) by creating a provider-purchaser split through the establishment of a new institution, the National Health Security Office (NHSO) responsible for purchasing of health services, and its governing body, the National Health Security Board (NHSB), responsible for setting policy, rules and guidelines and making decisions on the benefits package and on appropriate provider payment methods [30].

Overall, the reform has been considered a major success and a number of documents detail the achievements of UCS and the UHC reform over time. In particular, Evans et al. (2012) stress how the first decade of its implementation achieved improved access to essential health services for Thai citizens, especially for the poor; decreased catastrophic expenditures and household impoverishment; and increased satisfaction of UCS beneficiaries and healthcare providers.

#### Indonesia

Historically, the country had an assortment of five distinct health insurance categories: (1) serving civil servants and military personnel, (2) catering to private employees, (3) for poor people provided by national government, (4) for poor people provided by regional governments, and (5) private health insurances. Prior to the implementation of JKN, over 74% of the population lacked any form of health insurance coverage [31]. This subgroup was predominantly composed of informal workers, who constituted 54% of the population during that period [32].

As the constitution was amended to ensure the provision of social security and the National Social Security Act (NSSA) Law mandated the availability of national health insurance, in 2014 Indonesia initiated the UHC program called JKN. JKN is operationalized by Social Security Implementing Agency (SSIA) called BPJS Kesehatan. The Law 24/2011 on SSIA changed the management of the various insurance into a single public entity. JKN operates as a non-profit social health insurance program, with mandatory participation for the entire population, financed through participant contributions [33]. The government provides premium subsidies for the economically disadvantaged segment of the population. Central to JKN is the principle of *gotong royong* (mutual support), wherein individuals in good health support those in need of healthcare, the young support the elderly, and the rich help the poor. In January 2014, more than 121 million participants transitioned to the JKN. They were originally the members of previous schemes (civil servants, the poor, military personnel, and some part of formal workers) [34]. Presently, JKN provides coverage to over 260 million participants, constituting approximately 96% of the population [35].

Participants in the JKN programme are bound by established procedures, including for example initial consultation at a public or private primary healthcare facility. Hospitals may be accessed following a referral from the primary healthcare facility, unless the situation pertains to a medical emergency. The primary healthcare facilities, functioning as gatekeepers, are mandated to manage up to 144 primary diagnoses, thereby serving as a critical mechanism for ensuring quality assurance and cost control within the system. In general, JKN offers a comprehensive range of benefits, encompassing basic outpatient services and extending to catastrophic provisions, such as hemodialysis and cardiac surgeries.

The number of health facilities collaborating with BPJS Kesehatan has increased over the years. In 2014, more than 15,000 primary health care facilities, comprising 9,000 public primary health care (Puskesmas), and 6,000 private clinics, general practitioners, and dentists [34]. Currently, the number of contracted primary care facilities has surged to almost 24,000 [35]. In the context of hospitals, the number has expanded from 1,700 in 2014 to 3,000 in 2023 [34, 35]. In the JKN era, MOH continues to play a significant role in supporting the implementation of JKN towards UHC. The MOH is responsible for the formulation of clinical guidelines, setting technical norms, and allocating funds for subsidizing the poor.

The implementation of the JKN programme has also introduced a strategic purchasing approach aimed at improving the overall performance of the healthcare system. Within this framework, primary healthcare services are remunerated through a capitation scheme, which is calculated based on the number of registered participants at a given facility. In contrast, hospitals operate under the Indonesian Case-Based Groups (INA-CBGs) payment model.

Numerous research studies have been conducted to evaluate the efficacy of JKN programme in increasing population health outcomes. The JKN programme has been associated with improved accessibility to healthcare services, particularly inpatient and outpatient services, increased quality of care for maternal health services, as well as reducing healthcare inequality [36–38]. However, some improvements are still needed to leverage the benefits of JKN in Eastern Indonesia where the availability of healthcare service providers remains constrained [37].

### Context of health financing reforms

In this section, we provide information on the broader context and background to the health financing reforms in terms of the economic, political and health contexts in which they occurred (see also Box 1 and Table 1 for summary indicators by country). We focus in particular on the shock or crisis that have affected each setting to reflect on their direct and indirect influence on the health financing reform processes.

#### Nepal

Nepal has passed through a prolonged period of political transition since 1996, from a monarchy to a multiparty democracy, marked by armed conflict, ethnic protests, and frequent changes in government during the last two decades. Key political events included the civil war with Maoists, peace settlement, new constitution, and federalisation. Nepal was also hit by natural disasters, such as the major earthquake in 2015.

Nepal continued to face high levels of poverty (Table 1), though it has been moving out of least developed status. Nepal’s proposal to graduate from the Least Developed Country (LDC) category was endorsed by the United Nations General Assembly 2021 [39]. Per capita GDP, tax revenues, and government expenditure has been growing, however Nepal’s economy has become highly dependent on remittances, which was a major challenge during the COVID-19 pandemic [40–42]. Health, one of the strong contributors in the development pathway, also played important role in pulling down poverty in the country [43]. With the reduction in multidimensional poverty from 30% to 17% (2019) in five years, health has the lowest contribution to overall poverty given the low incidence of child mortality among the poor [44]. However, relative to other countries, health remains an important source of poverty.

Following the Comprehensive Peace Agreement (CPA) in 2006, health became a common agenda of the political parties. The constitution of 2015 institutionalised a rights-based approach to health, formulated policy to increase state investment in health, and steered the process for federal restructuring.

While Nepal’s spending in health has increased over the years, it is much lower than the countries in the region and health financing has remained dependent on OOP expenditure. With an average growth rate of 6% per annum (over 20 years- in constant value), per capita current health expenditure in Nepal has reached to US$53 in 2019 [40]. In 2017, the government health expenditures represented around 1.3% of GDP, while the same figures for low-income countries and the region were 2.8 and 2% respectively [45]. In 2000 to 2019, OOP spending has been over 50% of total health expenditure [46].

External actors have played a significant and growing role in the health sector. Within the framework of the Sector Wide Approach (SWAp), which was launched in 2004, the share of External Development Partners (EDPs) in national health budget increased from 21% in 2002/03 and reached about 50% in 2006/07 and 2007/08, later dropping to 28% in 2024/25 [47].

Most health and development indicators for Nepal’s population of 30 million have shown progress, with the incidence of poverty, for example, falling from 47% in 1990 to 20% in 2022 [48]. However, there have been long-standing concerns about maternal health, with maternal mortality ratios per 100,000 live births estimated at 539 in 1996, though now somewhat reduced to 329 in 2018 [44]. Within the health sector, maternal health was established as the common agenda of the government and the development partners in the mid-2000s. This led to the introduction of demand side financing to promote institutional delivery, as described above.

#### Thailand

During its modern history, Thailand has alternated periods of managed democracy, with continuing strong role of the monarchy, to others of military rule. A major turning point was the 1997 Constitution (the “people’s constitution”), which was the first to be drafted by a popularly elected Constitutional Drafting Assembly and entailed a reformist approach and renewed attention to civil liberties and public participation. This had important effects on the political and cultural context and influenced the results of the 2001 elections, paving the way for social reforms, including in the health sector [49].

However, the struggle to get the Constitution passed became intertwined with the Asian Financial Crisis (AFC) of 1997. Thailand was one of the fastest growing economies in Asia between 1960s and 1990s, but experienced cyclical crises including the 1997 one, which required that the country relies on an International Monetary Fund (IMF) support package to ensure recovery. The country completed the repayment of loans in 2003, but it took 10 years to recover from the crisis so that Gross National Income (GNI) per capita in 2006 was the same as that in 1997 [49].

In the 2001 elections, the newly formed, populist Thai Rak Thai (TRT) Party led by Thaksin Shinawatra proposed policies that appealed to the mass electorate, including a radical health financing reform and won with a large popular mandate [50]. Since 2001, the political history of Thailand has been dominated by the rise and fall from power of former Prime Minister Thaksin Shinawatra, and conflict over the rising military influence in politics [51].

Other critical events that occurred in Thailand during the 2000–2020 period include:

- The conflict due to the separatist movement in three South Muslim-majority provinces, which significantly worsened since 2004 but declined after a peak in 2010. Almost 7,000 people are estimated to have died and the conflict is still unresolved;
- The 2004 Indian Ocean earthquake and tsunami caused over 5,000 deaths;
- Floods in 2011 resulted in economic losses estimated at US$46 billion;
- Thailand was one of the first countries affected by the global COVID-19 pandemic in 2020. While it was relatively successful in containing the virus, its tourism-dependent economy was badly affected [9].

Despite political changes and instability, health has been a constant political priority in Thailand since the 1970s and has enjoyed continued political commitment. This has resulted in significant investment in health infrastructure and in particular primary health care, district and provincial referral hospitals, as well as support to a functioning healthcare workforce, ensuring rural retention through multiple strategies [27, 52]. During the decade of the 2000s, Thailand introduced several major health financing-related reforms to ensure a continuous gradual expansion of the health insurance coverage.

In terms of health financing, there has been a clear trend of increased public expenditure (general tax revenue) and a corresponding reduction of OOP expenses, from 34% to 9% of total health expenditure over 2000–2019, corresponding with the achievement of UHC in 2002 [53]. Development assistance has remained negligible overtime. Achievement in financial risk protection is evident by a noticeable reduction in the number of non-poor households being impoverished by health payment. Curative expenditure dominates total health spending, at about 70% of total [27].

#### Indonesia

Following World War II, Indonesia declared its independence from the Netherlands. The *Undang-Undang Dasar* (UUD) 1945 became the constitution and the primary source of state law. At the onset of its independence, Indonesia grappled with formidable political and economic challenges [54]. The political landscape was challenged by the large and diverse landscape, lack of cohesion, legacies of colonialism and separatist movements [55]. Concurrently, Indonesia’s micro and macroeconomic conditions faced stagnation, characterized by soaring inflation rates, an economic blockade, and an empty state treasury [56]. By 1950, political stability and a more consolidated Indonesian government emerged, particularly with the establishment of the Unitary State of the Republic of Indonesia [57].

The evolution of democracy in Indonesia has been very dynamic. From independence to the present, the country has experienced at least four distinct forms of democracy, each marked by upheavals and conflicts [58]. Firstly, there was parliamentary democracy (1945–1959), modelled on the Western concept, where parliament played a fundamental role in the government. However, this form of democracy was deemed less suitable for Indonesia due to the nascent democratic culture. Secondly, guided democracy (1959–1965) emerged, designating the first President Sukarno as the paramount leader in both democracy and revolution. The third phase was Pancasila democracy, marked by a leadership shift from President Sukarno to President Suharto, who then ruled for 32 years amid irregularities such as unfair general elections, restricted political freedom for civil servants, limited freedom of expression, a constrained party system, and widespread instances of collusion, corruption, and nepotism. The last phase is the democracy reform since 1998, triggered by the AFC in 1997/98. This era aimed to reinstate the fundamental principles of democracy, including direct general elections, freedom of the press, decentralization, regional autonomy, protection of citizens’ basic rights, and inclusive political recruitment.

Before being hit by the AFC, Indonesia had achieved impressive gains in economic growth since 1970. Until mid-1997, GDP grew an average of 7% a year, inflation was controlled, and the poverty rate fell to 11% [59, 60]. However, the crisis during 1997/98 was characterized by a fall in GDP of 13%, the collapse of the exchange rate against the US dollar, high inflation, and the poverty rate soaring to 27% [60, 61]. Around the same period as the AFC, Indonesia was also facing a series of natural disasters in rapid succession. These included widespread rice harvest failures in numerous regions due to an extended and intense dry season, infestations of crop pests, and extensive forest fires in Kalimantan. Additionally, mid-May 1998 witnessed riots that swept through many cities, leading to the ousting of President Suharto. Subsequently, this tumultuous period saw the separation of East Timor Province from Indonesian territory.

The AFC also had a direct impact on health financing and health service utilization in Indonesia. At the government level, the crisis reduced government expenditures for health care by 9% and 13% in 1996–1998 and 1998/99, respectively [62]. This declined resulted in shortages of pharmaceutical supplies, such as antibiotics and contraceptive pills [63]. As many households experienced a reduction in their purchasing power during the crisis, access to health facilities also became challenging. Households reduced the share of their budget spent on health services and increased their spending on food [63]. In addition, a significant decrease in utilization of government and private health facilities was also identified [62].

Like other countries in the region, Indonesia is experiencing an epidemiological transition, shifting from communicable diseases to an increasing burden of non-communicable diseases (NCDs) [64]. However, the maternal mortality rate continues to be among the highest in SEA and the poverty headcount remains at 10% in 2020 (down from 15% in 1990) [65–67].

Health financing data show an increase in Current Health Expenditure (CHE) from US$16 per capita in 2000 to US$133 in 2020. Out-of-pocket spending also continues to decrease from 45% in 2000 to 32% in 2020 as more people joined the JKN program. In 2020, the government contribution to CHE rose sharply from 30% in 2000 to 55% mostly for the COVID-19 prevention and care. Moreover, Government of Indonesia also increased its spending to pay the subsidy for the poor in the JKN program. In 2023, more than 96 million of poor people in Indonesia was subsidized to participate in the JKN program.

### Stakeholder positions and influence

In this section, we focus on the stakeholder roles, their positions in relation to the reforms and the degree of influence that they had over their introduction and implementation. We then move on in the discussion to the analysis of the strategies used by reform leaders to further the reforms. We summarise the findings using the categories proposed by Campos and Reich (2019), including the change teams, political leaders, budget and finance-related groups, bureaucrats, beneficiaries, interest groups and external actors.

#### Nepal

As summarised in Table 3, in Nepal there was strong political commitment from the major political parties in mainstreaming social protection, including investment in maternal health. This was driven by their political interests and previous commitments during the election period [10]. For example, the Communist health minister in the coalition government of the Nepali Congress and the Communist Party of Nepal-United Marxist Leninist (CPN-UML) in 2004 used their power to introduce the SDIP, which was previously committed to in their party manifesto. The Maoist government was highly supportive of maternity care for leftist ideological reasons. The party also wanted to gain more female votes in the upcoming parliamentary election. All political parties promoted a rights-based approach as per the CPA. This was supported by external development partners, who mobilised technical and financial resources in support of the sector (after initial resistance by some partners, due to concerns about sustainability) and specifically its focus on maternal health. Local democracy, as the system moved to a more federal approach, also promoted the health agenda and specifically women’s health. In addition, Non-Governmental Organizations (NGOs) advocated for maternal health and women’s rights, and some had strong connections to political leadership.

**Table 3:** Summary of key stakeholders’ interest and power, Nepal

Stakeholders category	Stakeholder group	Interest / Position	Power / Influence
**Change team**	A cohesive change team did not exist, but there was a body of experts from government entities, multilateral and bilateral agencies, Civil Society Organizations (CSOs) & NGOs, subject experts, who individually played an influential role - having commitment and providing advice and ideas on the *Aama* programme (free maternity care) and driving the reform agenda, with some changes in actors over time.		
**Political leaders**	Maoist-led government	Highly supportive as it was a leftist party with an ideology of state-funded health care.	Highly influential as the party was in power and holding key posts (including in health)
**Bureaucracy**	MoH and Population	Committed but with some internal differences on political ideology, design and implementation.	Power was high as it played a key role in designing the reform agenda and leading its implementation.
**Budget-related groups**	MOF; National Planning Commission (NPC)	-Position of the MOF was supportive with the condition that donors would fund in the initial years. - The position of NPC was supportive but with concerns on financial sustainability.	-Power of MOF was high as they set the budget ceiling for the health sector. -For NPC, influence was moderate; its main role linked to reaching the MDG targets.
**Beneficiaries**	NGOs + CSOs	Supportive in advocacy for reform and political networking	Moderate power but played a critical role through advocating with policy makers and concerned stakeholders for investment in maternal health and acting as main liaison on safer motherhood.
**External actors**	Bi- and multilateral organizations	-World Bank initially concerned for financial sustainability, was pro private sector engagement but later supportive of reforms. -WHO supportive of reforms but less engaged in the process. -Department of International Development (DFID) highly supportive of the reforms. -USAID supportive of reforms with preference to onboard private sector and for micro planning at local level.	DFID is moderately powerful as the funding agency and provider of key TA to the reforms. Overall high financial dependency on EDPs in the health sector gives policy influence.
**Interest groups**	Nepal society of obstetricians and Gynecologist (NESOG); Hospital Development Board	-Hospital development board was supportive mainly in advocacy and lobbying; saw reform as revenue generating scheme for the hospitals. -NESOG was supportive of the scheme but concerned over mismatch between human resources and potential increase in workload in hospitals leading to poor quality of care and that financial incentives with poor monitoring might trigger too high caesarean section rates.	Moderate as the professional bodies advocating for quality maternity care and as provider of the care.

Source: [10]

#### Thailand

There is a broad consensus in the literature that the UHC reform in 2002 was driven by the convergence of political commitment, civil society mobilisation and technical experience and knowledge [28]. However, all analysts stress the key role played by a group of like-minded “reformists” in the MOPH and at the Health Systems Research Institute, who had been systematically documenting health inequities and developing evidence-based policy options to tackle them, including radical financing reforms to achieve universal coverage [28] (Table 4). In 2000–2001, they were able to take advantage of the climate of reform to present the UCS proposals to the TRT Party leader and gain his support. In addition, the change team also developed strong ties with Non-Governmental Organizations (NGOs) and civil society organisations, and in turn, civil society played a key role in securing Parliament’s commitment to UHC.

**Table 4:** Summary of key stakeholders’ interest and power, Thailand

Stakeholders category	Stakeholder group	Interest / Position	Power / Influence
**Change team**	Group of like-minded professionals with rural doctor background who held influential roles within the MOPH and other public bodies.	- strong support for creation of UCS and introduction of a universal, tax-funded system	- strong political links with TRT party - bureaucratic resources linked to leadership position in key public institutions; moved within existing bureaucratic mechanisms to achieve progress with their agenda - well respected technical expertise, knowledge and experience, but also politically savvy - capacity to mobilise international and civil society support
**Leadership**	Head of State, President, Prime Minister, Prime Minister’s Office, Parliament	- support to radical social reform, including to creation of UCS - creation of UCS included in political manifesto before elections	- large majority in Parliament and popular support after 2001 elections which allowed for new parties
**Bureaucracy**	MOPH (key role of Permanent Secretary)	- leadership broadly supporting UCS - some (little) internal opposition, especially initially	- managed the agenda directly - political appointees with technical experience/knowledge
	National Health Security Office	- created by the UHC reform/UCS introduction and strongly supportive	- technical experience and politically supported
	Health Systems Research Institute	- strong support for creation of UCS	- able to provide evidence-based, technical support to different reform elements, also based on experience from other funds.
**Budget-related groups**	Minister of Finance, Bureau of Budget	- interest in reducing overall health sector budget, no clear position on creation of UCS	- reduced power as not responsible for negotiations due to capitation-based budget - funding decision for financial gap taken by government at higher levels (Prime Minister)
**Beneficiaries**	Citizens, civil society organizations	- strong support for creation of UCS - well organised and also mobilised by change team	- empowered by social shift and increased role for civil society in 1997 Constitution - power through ability to create awareness, advocate, raise funds and signatures - direct, close links to change team, with reciprocal support
**External actors**	Multilateral organizations	- initial opposition of World Bank due to financial concerns - strong support from WHO and ILO (mobilised by change team)	- influence in political/technical debate, but external funding is limited (therefore power is relatively low) - strong support from some organisations counterbalanced opposition of others - personal links between change team and supporting external actors
**Interest groups**	Medical associations Public/private healthcare providers	- mostly opposed to the reform	- some influence through Parliament, but overall lacking the access to the resources that the change team had and less well organised/mobilised.

Source: [9]

With political and broader societal backing secured, the change team and technocrats took the lead. To formulate specific policies, the MOPH set up 10 working groups, each with representatives from the public health-care sector, consumer groups and private healthcare providers, and also in collaboration with many functional departments within the MOPH. A committee known as the “War Room”, chaired by the Deputy Minister of Public Health, was set up to coordinate and monitor activities pertaining to policy implementation and to solve emerging problems [28].

There is general agreement in the literature that the UHC reform was not slowed down by budgetary concerns. The decision to adopt an annual per-capita budget for the UCS took away much of the discretionary power of the Ministry of Finance’s Bureau of Budget that had previously done all the budget negotiations [68], based on an opaque “patronage system” (KII 1 Thailand). Efforts were made to create a transparent process that involved multiple stakeholders (e.g., representatives of public hospitals, private hospital associations, academia and NGOs), and was evidence-based, so that the Bureau of Budget became one among numerous other actors involved in the process. This initially generated substantial tensions but was accepted in the longer term [68].

The financial sustainability of the UCS had raised questions from some opposition groups as well as international experts [28]. The World Bank in particular raised concerns that the reform was not sustainable in the longer term due to unpredictability of future costs and too small fiscal space in the context of the recovery from the AFC. It also feared it would bankrupt public hospitals and dilute quality of services [69]. The change team used their links with other international organisations and in particular WHO and the International Labour Organisation (ILO) to counter this criticism and provide greater legitimacy to the reform [69].

Other groups opposed to the reform included the medical profession and private health providers. Doctors in particular were concerned by the system of complaints and malpractice that was being set up and the liability this provision would generate, while private hospitals were concerned about cost sustainability. The change team managed these concerns by dropping the complaints system and ensuring a rapid approval of the reform before opposition could consolidate. In addition, it is noted that the opposition groups lacked the power and resources that the change team had, in terms of political, social and international links [69].

There was also some opposition from within the MOPH, mostly because the reform had changed the internal structure by introducing a provider/purchaser split and redirected most of the MOPH budget to the NHSO. The difficult relationship between NHSO and some within the MOPH resulted in attempts from MOPH “conservatives” to slow the pace of reform and was worsened by critical and adversarial NHSO statements [28]. Only strong political leadership and the fact that the MOPH leadership was mostly in favour of these reform stopped the MOPH conservatives derailing the reform [69]. A change in tone by the NHSO also helped to reduce opposition.

#### Indonesia

Studies suggest that the success of putting the NSSA law in place was largely due to the leadership of President Megawati [70, 71]. President Megawati used her power and authority to establish a task force to promote social security issues both in government and in the public arena. With this task force, President Megawati formed strategic alliances to prepare draft legislation and academic papers for the NSSA. During this time, members of the task force worked hard not only to prepare the bill, but also to educate the public, the private sector, and even the government and line ministries about the importance and benefits of having a social security system in place. Nonetheless, there were also many conflicting interests from the private sector, business associations, foreign investors, international organisations, and even from within the government itself, who were opposed to the law being enacted. Fears that the forthcoming social security law would hamper the business of private insurance companies, reduce business profits, and disrupt the state budget were among the reasons for opposition. In dealing with the groups that opposed the draft of NSSA law, the task force team had to negotiate and make some compromises, particularly on the issue of premium contributions.

Some tensions between the MOH and other ministries, such as the Ministry of Finance (MOF) and the Ministry of State Enterprise, also occurred concerning program administration, membership, and the roles of local government in providing and administering health services [71]. Under the leadership of Minister Nafsiah Mboi (June 2012-October 2014) the support of the MOH towards the social health insurance scheme was more apparent. She demonstrated strong attention towards the scheme and prioritized finalizing the conceptualization and planning for implementation of JKN, while also aiming to expand healthcare facilities and strengthen the capacity of healthcare providers through continuous training [72].

Six working groups involving various people from the health sector and domestic and international experts were established to address key challenges in implementing the JKN. Members of the working groups came from, among others, the Indonesian Medical Association, Health Economists, representatives from the MOF, the National Planning Board, and other organizations. The working groups were set up to ensure the timely and smooth launching of JKN in early 2014. The MOH also participated in designing the social health insurance, together with the NSSA task force team and parliament members [71].

After Megawati stepped down, political momentum slowed, especially during the first period of Yudhoyono’s presidency. Other priorities intervened, and there was a lot of pressure on the government from those who did not want the law to be implemented because they would lose direct access to the social security funds administered by the four state social security companies, as well as pressure from private insurance companies which were concerned about losing their market.

After more than ten years after the launch of the NSSA Law in 2004, the JKN was successfully implemented in January 2014, thanks to continued pressure and support from a variety of stakeholders (Table 5), including parliamentarians, international organisations, and academics. Parliamentarians played a particularly crucial role: their engagement extended to design contributions and urging the government to expedite the enactment of the SSIA law before the stipulated timeframe in the NSSA expired.

**Table 5:** Summary of key stakeholders’ interest and power, Indonesia

Stakeholder Category	Stakeholder Group	Interest/Position	Power/Influence
Change team	Group of people with various background such as academics, NGO (KAJS), and parliament members	Strong support for development and creation of social security, including social health insurance. The change team are also part of other stakeholder groups; hence their roles, power, and influence are described below	
Leadership Politics	President	In the run-up to the NSSA law that laid the foundations in 2004, President Megawati had strong commitment and interest in social security issues and was very keen to pass the NSSA bill immediately. In the second phase, the newly elected president did not have a strong interest in health issues. He delayed the stipulation of SSIA Law although it was mandated by NSSA Law.	The President utilized her power to form a special team consisting of academics specializing in social security issues (post 1998 crisis)
	Parliament	The members of political party (PDI-P) strongly supported the President and her decision. Parliament members (especially Commission on Health) had strong interests in the stipulation of the NSSA and SSIA laws, thus they initiated the discussion process in the parliament.	Parliament had a strong commitment and influence to push the government in passing the bills. They also proposed the social health insurance concept to the government. As the government seem to lack interest on the social security issues, the parliament members then used their power to initiate the draft bills discussion in the parliament, involving civil society organization and academicians
Bureaucratic politics	Ministry of Health	Support towards the universal health scheme and its implementation	Had limited power/influence outside the ministry but held negotiations with MOF to set up premium contributions, provided technical guidance for SHI, and established working groups to address implementation challenges
	Ministry of Labour	Initially did not support the social insurance scheme due to competing interests (especially in ensuring the welfare of employees)	Suppressed the passing of the NSSA bill, especially when there was discussion of equal share of contribution by employers and employee.
	Other ministries (Ministry of Planning and Development Plan, The Coordinating Ministry for People’s Welfare, National Social Security Council)	Supported the creation of social health insurance by providing required policy instruments	Provided policy instruments required to create SSA and implement JKN
Budget-related groups	MOF	Strong interest to keep the state contribution to a minimum	MOF has influence over state budget, Coordinated with line ministries on the contribution fee from the government for poor group
Beneficiaries	Citizens, civil society Organisation (KAJS) Academics	- KAJS: in favour of SSIA law. Provided strong support towards the stipulation of the bill. - Some labour unions were supportive, while others opposed the social security reform because they did not understand that it would bring them more benefits than harm - Academics generally supportive - Business association opposed the plan as they think it would put more burden on the business, thus reducing business profit.	Civil Society (KAJS) mobilized people; workers’ union, urban poor consortium to fight for SSIA to be stipulated and implemented by taking the matter to court for judicial review Task force (comprised of academics) had a strong influence on Megawati in terms of designing the system and draft law and also conducting awareness raising of the public
External actors	International organisations	Support the establishment of the social security system	Has power in terms of providing knowledge about social security system and being able to fund studies, facilitate discussion and public/social dialogue
Interest Groups	Health workers’ association Employers’ association Labour Union	Health workers’ association provided technical analysis to support SHI implementation Some labour union and professional association opposed the reform (reluctant to pay for workers’ contribution)	Try to influence and negotiate with the government. Delaying the implementation of mandatory contribution by employer

Source: [11]

The establishment of *Komite Aksi Jaminan Sosial* (KAJS/Social Security Action Committee) as a civil society response to the delay from the government was significant in pushing the social security reform. The committee comprised of dozens of national labour unions, professional/employee unions, and various social movements, including representatives from farming, fishing, students, and poor urban communities. KAJS serves as the overseeing body responsible for monitoring the implementation of NSSA Law. The committee played a pivotal role in steering the formulation of the SSIA Bill by actively engaging with the government during that period. Employing a multifaceted approach, KAJS actively advocates for the advancement of the SSIA agenda through channels such as dialogues, marches, deliberations, and public demonstrations, and in certain instances, through legal actions taken against the government to expedite the implementation of social health insurance. Substantively, KAJS benefited from the support and guidance of academics and international organizations. During this period, the support for SSIA was far greater than those in opposition. Opposition primarily came from a minority faction of workers/employees and employers’ associations.

### Strategies used by change teams to move forward reform

In this section, we examine the strategies used to move forward the reforms in the three cases.

In Nepal, the ‘change team’ was more diffuse, compared to Thailand, but a number of similar strategies are highlighted by the case study, such as the importance of evidence generation, facilitation of policy dialogue between stakeholders, generating support from influential donors, and creating legitimacy through a rights-based approach, grounded in the safe motherhood movement (Table 6). Indonesia’s reform underscores the strategic influence of the executive, as well as organized civil society in supporting political momentum of reform.

**Table 6:** Summary of key strategies used by actors to support reforms in Nepal

Stakeholders category	Political economy factors and dynamics	Strategies used by ‘change team’	Practical actions / outcomes
**Leadership politics**	• Free health care was incorporated as a component in the CPA with political consensus • Leaders of all national political parties (alliance of seven political parties and Maoists) supportive of social sector reform	• **Coalition building**: Seized opportunity to deliver peace dividend in changed political context in favour of reform by engaging new political leaders. • **Evidence and information**: Used synthesized evidence to justify reform agenda	• The political parties manifesto incorporated the reform agenda. • Consensus among national political parties on health being incorporated as a basic right in the interim constitution
**Bureaucratic politics**	• General support at the top level of (Ministry of Health and Population (MOHP) but level of proactivity differed, mainly depending on the senior officials’ political interest and sustainability of the proposed reform. • Highly supportive based on evidence synthesis but subject to ministerial approval	• **Evidence and information**: Closely worked with supportive partners and technical assistance providers in generating evidence for the proposed reform. This was continued over time as the policy went through a series of iterations and expansions. • **Enhancing the legitimacy of policy**: Advocated the leaderships as the strategy to achieve international commitment of MDGs and national goal of poverty reduction • **Increasing organizational strength of supporters**: Led the discussion towards establishing the SWAp	• Proposed roadmap to reform with implementation strategies. • Costed plan for nationwide scale up of reform which worked as the basis for resource framework to secure domestic and international funding.
**Budget politics**	• Sector wide approach adopted to harmonize donor support and expand fiscal space for the health sector. • MoF supportive when policies backed by external funding • Concerns for sustainability, especially for broader free care policy • Maternity care and free care programme evolved as the priority programme for national planning commission and MOF, which implied higher priority in budget allocation	**Increasing organizational strength of supporters** • Pool funding mechanism implemented, which allowed flexibility in the operationalization of donor fund through the government treasury. • Donor made strong commitment to finance the initial phase of the implementation of the reform agenda.	• Consensus reached on progressive increment of domestic financing for sustainability • Provision of earmarked funding for the reform. • Joint financing agreement among government and major development partners.
**External actors politics**	• Actors varied from highly supportive to skeptical to the reform • Some were mainly concerned about fiduciary risk and financial sustainability	• **Evidence and information**: Evidence synthesis presented among EDP groups for consensus building • Mobilized technical assistance programme to closely interact with government counterparts to get government buy in • **Coalition building and consensus building**: Mobilized global networks such as the International Health Partnership, P4H • **Persuade opponents to weaken their position**,** by adding desired goals or mechanisms**: Among pooled partners, supportive actors engaged actively to prevent opposition of non-supportive actors, by meeting some of their concerns in the policy implementation plan.	• Committed technical assistance towards reform. • Securing donor funding for initial years for program implementation • Multiple studies and assessment advocating for evidence-based decision making and programme harmonization
**Beneficiary politics**	• Frequent disruption of public services due to political conflicts led to frustration among general public towards the actors, systems and services. Agenda of social inclusion and women’s empowerment made them somewhat vocal on their issues	• **Encouraging participation**,** transparency and accountability**: Community empowerment and demand creation through mobilization of Female Community Health Volunteers (FCHVs), mother groups	Supportive of the reform, contributing for success
**Interest groups politics**	• Aftermath of civil war, growing public expectations on basic services, increased role of civil society in shaping agenda for social reform • Growing private sector in urban setting, attracting patients. • Private sector, particularly medical colleges, became part of the scheme, however they were concerned about the reimbursement rates.	• **Evidence and information**: Evidence generation on supply side barriers and used evidence to advocate service strengthening. • **Mobilize supporters** in groups and communities: Organizational empowerment of civil society • **Encouraging participation**,** transparency and accountability**: Implementation of social audit, community hearing through engagement of civil society/NGOs to help promote accountability • Enhanced transparency and accountability through citizen charter in public service provision • **Coalition building**: Coalition of civil society organizations engaged with parliamentarians and political leaders to secure RH rights	• Contribution of civil society organization to draft bills on RH and safe motherhood, safe abortion, skill birth attendant policy • Selective private sector providers were brought on board, supporting expansion of coverage. However, the larger segment of the private sector remained out of the scheme. • Higher rates of reimbursement by the health insurance scheme in comparison to the maternity scheme later provided more attractive option for private providers. membership.

*Note: This analysis is primarily on the free maternity care policy* [10]

In Thailand, the critical role of the reformist group at the heart of the change team emerges (Table 7). This change team identified the political opportunity for the reform and then mobilised essential resources, at the bureaucratic, political and social levels, and put in place effective strategies to ensure progress of the reform [69]. The strategy used by the change team has been called “the triangle that moves the mountain”, because of the synergistic and interlinked mobilisation of civil society and public support, political support and use of evidence and technical experience [28, 73, 74].

**Table 7:** Summary of key strategies used by actors to support reforms in Thailand

Stakeholders’ category	Political economy factors and dynamics	Strategies used by change team	Practical actions / outcomes
**Leadership politics**	Leaders of new TRT party supporting ideas for radical social reforms.	- **Coalition building**: Persuade political candidates or elected officials in the legislature or executive to adopt your issue, through personal meetings, position papers, or political incentives. - **Enhance the legitimacy of policy**, by connecting it to positive social values.	UCS included in political manifesto before elections and supported after electoral win.
**Beneficiary politics**	In the context of democratisation and increased relevance for popular participation, as well increased need for financial protection, civil society organisations and NGOs become more relevant and can be mobilised in support of UHC/UCS reform.	- **Coalition building**: Build a coalition of supporting groups or players, with a recognizable name and sufficient resources. - **Increase the organizational strength of supporters** by providing increased material resources or by providing experienced staff or fostering political skills. - **Mobilize supporters** in groups and communities in public demonstrations to call for action. - Provide **information and evidence** to supporters, including technical and political information.	Civil society organizations and NGOs mobilized and supported with funds and technical information to prepare their draft of National Health Security Act and gather signatures in the petition campaign.
		- **Encourage participation**,** transparency and accountability** as a strategic approach to muster the support of civil society organisations, patients’ groups, etc. - **Change the decision-making processes** (e.g. through public hearings) in order to expand the number of supporters.	Active participation of civil society in designing and implementing UCS via the National Health Security Board, as well as establishment of National Health Assembly (2007).
		- Persuade supporters to **strengthen their position**, by adding more benefits as an incentive.	Pragmatic adoption of a generous benefit package in line with existing schemes and despite budgetary concerns.
**Bureaucratic politics**	Overall supportive of UCS, but some opposition to UCS reform from some in the MoH. UCS emptying the MoH of much of its roles and power in favour of the NHSO.	- Meet with opponents to **seek common goals** or mechanisms, and thereby reduce the intensity of their opposition. - **Persuade opponents to weaken their position**,** by adding desired goals or mechanisms** to the policy.	Non-threatening language towards “conservatives” within the MoH and their interests is adopted by the change team (for example. by pointing to general objectives of the policy and not objectives directly threatening or opposing their practices). Opponents within the MoH are included in participatory decision making so that they are ‘co-opted’ to the reform cause and their opposition is minimized.
**Budget politics**	New ruling party wants to stress the difference with the past and abandon patronage system for budget allocation towards a more transparent one. Leadership politics prevails over budget politics, despite financial constraints created by the economic crisis.	- **Change decision-making processes**, in order to prevent some opponents from participating.	Bureau of Budget/Ministry of Finance is bypassed and budgetary decisions are made at higher levels by the Prime Minister and (later on) through a transparent, evidence-based and participatory process (i.e. definition of capitation levels).
		- Persuade opponents to **weaken their position by adding desired goals** or mechanisms to the policy.	Use of evidence and previous experience to design technical features of UCS so that it addressed budgetary concerns (cost containment, strategic purchasing, etc.)
**External actors’ politics**	Varying level of support from international organisations.	- Seek **common goals** or values with the non-mobilized, to persuade them to take a public position of support. - **Strengthen links based on similar ideals and personal connections** with non-mobilized, to obtain their support and counterbalance opposition.	Change team makes use of personal links with supportive international organisations (ILO, WHO) to counter criticism and increase legitimacy of the reform. At the same time, it marginalizes opponents in the international community (World Bank).
**Interest groups’ politics**	Opposition to the creation of UCS, in particular from medical profession and private health providers.	- **Encourage participation**,** transparency and accountability** as a strategic approach to manage tension with interest groups.	Interest groups are included in participatory decision making so that they are ‘co-opted’ and their opposition is minimized.
		**Change the decision-making processes**: **-** Rapid pace of reform to prevent opposition consolidating. - Change decision-making processes in order to prevent some opponents from participating.	Interest groups lacked power and resources of the change team and had not prepared for a long time to oppose the reform. Change team had more time to prepare and ensured rapid approval of reform, before opposition mounted or coalition of opposers formed.

Source: [9]

Another strategic approach used by the change team was the rapid pace of the decision-making processes. This “blitzkrieg strategy” allowed the change team to gain an advantage against opponents of the reform (for example, the medical profession) as their opposition would only come during the legislative process. Strategic choices and compromises also had to made to ensure the political and technical feasibility of reforms, including maintaining a generous benefits package (to reduce ‘losers’ from the new policy), maintaining the separate SSS scheme, agreeing on funding the new UCS policy from general taxes [28, 52]. When tensions emerged with opposition groups, they were managed through the change team’s strong political leadership and a change in tone towards collective problem-solving. In this sense, encouraging participation, transparency and accountability was also a key strategy to manage tensions with different constituencies (e.g., the private sector and medical professions) and to muster the support of others (e.g., civil society organisations, patients’ groups, etc.). In other occasions, the change team made a strategic use of evidence, explicitly drawing on it to inform or support design choices and mobilising international support through personal networks and publication of results of the UCS, creating a narrative of success that would help protect achievements.

The Indonesian case study highlights a number of similar strategies to those deployed in Thailand and Nepal, including the key importance of mobilising political support, including from the President but also key parliamentary groups. Comprehensive generation of evidence by diverse stakeholders, supported by international organisations was also important, building on earlier reform experiences (Mboi 2015). Other strategies (Table 8) including coordination with diverse groups, establishing policy dialogues, public awareness raising, and use of legal challenges when implementation under President Yudhoyono was lagging. The policy design also drew on national values of mutual support and took urgency from the public exposure to risks highlighted during the 1997/1998 AFC. Engagement of civil society, notably through the KAJS, and its alliance with parliamentarians, were key: during the law-making process, KAJS closely monitored the special session in the parliaments, attended almost every meeting, had a special seat in the discussion room and could comment and send feedback directly to Committee IX members who were present in the discussions [11].

**Table 8:** Summary of key strategies used by actors to support reforms in Indonesia

Stakeholders category	Political economy factors and dynamics	Strategies used by ‘change team’	Practical actions / outcomes
**Leadership politics**	• During the reform period, there were three presidential changes. The first two presidents strongly supported social security reforms. President Megawati seized this opportunity to enhance her electability during the presidential election • Meanwhile, during the Yudhoyono era, the reforms were delayed. Frictions between President Yudhoyono and his predecessor contributed to the postponement of various policies, particularly the implementation of social security initiatives.	• **Coalition building**: Built coalitions with NGOs and parliament members to seize opportunities for advancing the NSSA law.	• Amendment of the 1945 Constitution • Formation of a team for developing the social insurance concept and drafting the NSSA Law
**Bureaucratic politics**	• Various Ministers of Health displayed differing viewpoints on the universal health scheme and its implementation. One of the Minister of Health did not show interest in the newly stipulated NSSA law and created a new system instead, which was controversial. • Other Ministries of Health supported the NSSA law and the establishment of the SSIA law.	• **Evidence and Information**: Synthesized evidence was used to justify the reform agenda.	• Establishment of six working groups involving various stakeholders from the health sector, along with domestic and international experts, to address key challenges in implementing the JKN. • The MoH, in collaboration with the NSSA task force team and parliament members, proposed the design of social health insurance.
**Budget politics**	• The Ministry of Finance (MOF) supported social health insurance but was highly concerned about the contribution fees for the poor, as these would be funded from the state budget.•	• Strategic dialogue to **seek common goals or values**: the MOF participated in the team during the creation of the NSSA and SSIA laws and in the conceptualization of the JKN.	• A consensus was reached on the contribution amount for the poor
**External actors politics**	• All the external actors mentioned supported the establishment of the social security system	• **Seized opportunities provided by external actors’ politics** to learn about the evidence and information related to the social security system. • **Evidence and information**: Utilized this evidence to persuade other actors to support the implementation of social security reforms	• Provided committed technical assistance to support the reform process. • Conducted multiple studies and assessments advocating for evidence-based decision-making.
**Beneficiary politics**	• Initially, the beneficiary group was reluctant about the reform, as it appeared to do more harm than good. However, after being influenced by academics, they came to support the reform.	• **Mobilized supporters within groups and communities** to empower civil society organizations.	• Public demonstrations • Dialogues • Marches in support of the reforms
**Interest groups politics**	• Some labour unions and professional associations opposed the reform, as they were reluctant to pay workers’ contributions. • In contrast, health workers’ associations supported the reform.	• **Evidence and Information**: Generated evidence on supply-side barriers and used it to advocate for service strengthening.	• The health workers’ association provided committed technical assistance to support the reform.

Source: [11]

## Discussion

In this section, we reflect on the key themes emerging from the three case studies and how they compare, highlighting some similarities and differences of context, stakeholder and stakeholder management strategies, and how these elements influenced the reform trajectories.

### Strategic approach and composition of change teams

One point of contrast between the case studies was related to ‘change teams’ at the heart of the chosen focal reforms. In the Thai case, the change team emerged as key to the design, adoption and implementation of reforms. Technically strong, politically savvy and well-connected, the change team was ideologically committed to UHC but seen as politically impartial and free from conflicts of interest (KII 5 Thailand). The change team was a close-knit group with key contacts inside and outside of the MOPH. In Indonesia, the momentum came primarily from the executive. The change team comprised individuals from diverse backgrounds, including academics, civil society representatives, and parliamentary members. The academics were responsible for enhancing public knowledge and awareness of social security issues, including social health insurance. Civil society representatives focused on mobilizing public support through marches and demonstrations to pressure the government to accelerate the reform process. Meanwhile, parliamentary members worked within bureaucratic channels to advocate for governmental action. In both countries, the change team demonstrated not only their technical skills and knowledge, but also their experience in navigating bureaucracy and governmental politics and their capacity to mobilise different sources of power at political, societal and international levels and use effective strategies to move the reform agenda forward. They saw themselves (and were in practice) policy entrepreneurs or “match makers” between evidence and politics (KII 5 Thailand). By contrast, in Nepal, there was no clear change team, but rather a more diffuse set of actors supporting reforms over time, with more external influence, which may in part explain the more fragmented nature of these reforms.

### The role of political leadership

Political leadership played a key role in the Thai and Indonesian case studies, but less so in Nepal, where reforms were negotiated at a more bureaucratic level, which may link to their more gradual and partial nature. In Thailand, the alliance of President and reformists was key to the rapid deployment of the UCS, while in Indonesia, presidential engagement launched the first wave of reforms and had to be re-energised under a second president, thanks to an effective alliance of parliamentarians, civil society and academics.

### Windows of opportunity

In all three case studies, reform took place in a window of opportunity generated by political change, which was itself a product of a previous crisis (economic in the case of Thailand and Indonesia, and conflictual in the case of Nepal). Following Kingdon’s model [5], the opening of the window of opportunity was allowed by the alignment of three ‘streams’: the problem stream (deepened perception of difficulties for the public in access to healthcare and catastrophic expenditure in the aftermath of the financial crisis in Thailand and Indonesia; and poor maternal health indicators, embedded in a context of poverty and rural exclusion in Nepal); policy stream (technical solutions based on experience of other prepayment mechanisms and supported by research capacity and specific studies commissioned in all three settings); and political stream (new government and push for radical political change in all contexts, with political dividends envisioned from the reforms).

In the case of Thailand, this “window” to act was short-lived and the change team had to move quickly to take advantage of it. In Nepal, the fall-out of the CPA took longer to materialise, in the form of the new Constitution and the federalisation agenda, and the reforms that we track took place more iteratively and over a longer period, as was the case in Indonesia, where the reforms took place in two waves under different presidents, thanks to sustained support from significant groups including the Parliament and civil society.

It is also important to note how the change teams adopted (or not) strategies to take advantages of the windows of opportunity, for example with changes in the pace of the reforms. In Thailand, the decision making and implementation of the UCS reform in 2001/2 happened at a rapid pace, described as “blitzkrieg strategy”, which took advantage of the window of opportunity and avoided the consolidation of opposition to the reform [69]. This is a strategy that has been applied in other settings, for example in the United Kingdom for the reform of the internal health markets [75]. After that, reform progress returned to a slower pace during implementation that was gradual and incremental, and characterised by flexibility. The same time pressure does not seem to have been experienced in Nepal, which may be a reflection of the relatively less clear opposition from organised groups, as well as the less deliberate reform agenda (linking to the more diffuse ‘change team’ aspect highlighted above). In Indonesia, reforms were intense in phases, with lulls in-between linked to the absence of political leadership.

### Stakeholder mobilisation and communication

It is interesting to compare strategies used across the settings to move the reforms forward (Table 9). In all cases, use of evidence and information was very important, along with coalition building, and enhancing the legitimacy of the policy (for example, by drawing on the Millennium Development Goals (MDGs) and rights-based approaches in Nepal, and by connecting to traditional social values in Thailand and Indonesia). Mobilising support (for example, from civil society) and addressing opposition by meeting some of their demands was identified in all settings, but most significantly in Thailand, perhaps because of the greater potential opposition (for example, from beneficiaries of existing schemes which feared dilution of benefits, from the MOPH because of changes to its role, and from private providers).

**Table 9:** Comparison on actor management strategies across settings

Topic	Possible strategies to address these issues	Nepal	Thailand	Indonesia
Financing and ownership structure	• Making adjustments to reduce costs (e.g. ensuring budgets are capped, closed provider payments) • Leveraging additional support (including from international development partners, where appropriate and feasible) • Focusing on efficiency goals and their communication to stakeholders • Negotiating win/win outcomes with opponents who have influence • Making strategic concessions, if not conflicting with key objectives, to bring those potentially negatively affected on board; in many examples, this involves protecting the benefits of existing scheme members (e.g. in pooling reforms)	✔	✔ ✔ ✔ ✔	✔
Reform ‘windows’	• Identifying likely opportunities and preparing reforms in advance • Working with opposition parties, where power transfer is possible • Delaying or modifying reforms if the context is currently too hostile until a reform window opens		✔	
Historical factors	• Ensuring that the reforms address historical injustices and address/reflect national aspirations, contributing to reconciliation post-conflict, and being framed in this way	✔	✔	✔
Political factors	• Target reforms at relevant level that has responsibility for (and influence over) the relevant functions • Engage higher level political leadership in the policy development process, especially in centralized systems	✔	✔	✔
Decision making (agenda setting, design and adoption)	• Skill advocates in the key fora to represent the case for reforms • Ensure all key stakeholders across relevant agencies are consulted and briefed on the reform goals and rationale • Increase the participation of actual and potential supporters • Create or boost networks or coalitions that increase the effectiveness of supporters • Ensure that supporters can resource their activities	✔ ✔	✔ ✔ ✔ ✔ ✔	✔ ✔
Ideological and cultural factors	• Frame reforms in a manner consistent with dominant ideologies and values • Focus on elements which speak to positive values of nationhood, mutual support, equality, care for one another, protection of family	✔ ✔	✔ ✔	✔ ✔
Evidence	• Ensure supporters have access to credible evidence to support the reforms • Develop evidence and related briefs which are localized and specific to likely policy-maker concerns in relation to this policy • Data should be timely and accessible and presented in a variety of fora and formats • Evidence should offer positive solutions to the policy problem	✔ ✔ ✔ ✔	✔ ✔ ✔ ✔	✔ ✔ ✔ ✔
Global factors	• Supportive international development partners and civil society organizations should be mobilized to provide resource (ideological, technical, financial) to promote the reforms • Relevant global norms can be deployed, although these will need tailoring to the country’s context	✔	✔	✔
Accountability and oversight	• Expand spaces where supporters can influence and monitor the policy • Close-to-policy advice to support policy, design and monitoring	✔	✔ ✔	✔ ✔
Implementation	• Engaging in dialogue with providers and interest groups to increase awareness of policy, its intended benefits, and mobilise support • Setting up fora for regular review of policy and trouble-shooting, allowing for iteration to ensure problems are surfaced and addressed	✔	✔	✔
Equity	• Ensure that vulnerable groups are represented in consultative process • Mitigate losses and take actions to buy off the ‘losers’ from the policy • Policy design, iteration and communication to ensure equity goals are given prominence and priority	✔	✔ ✔ ✔	✔

In Nepal, the external actors (development partners) played a much more significant role, linked to Nepal’s weaker economic situation and greater aid dependency of the health sector in particular, so building consensus amongst donors assumed a more prominent role. The interaction with international players in Thailand differed – in this case, the change team drew on international learning early in the process, but also used international approbation to cement the reforms as the Thai experience was shared internationally and given a strong positive reaction – while in Indonesia, the external players mainly supported evidence generation in support of developing the reform design and adoption.

The Thai case study highlights overt political adoption strategies, including changing the decision-making processes (for example, shifting budget decision to the Prime Minister to bypass challenges by the MOF). Some of these, such as the establishment of the NHSB and later the National Health Assembly, not only increase supporters for these reforms but also likely change the margins of maneuver for future reforms. There was also more dialogue on reform content to bring people on board – seeking common goals, and reflecting opposition demands in some elements of the reforms. The overall key elements of all strategies in Thailand, according to one KII, were compromise and negotiation, as well as strategic use of participation (listening to and respecting others), which were integrated within the open recognition of the importance and power of policy dialogue in itself [9]. The approach to the policy dialogue remained flexible, adaptive, gradual and pragmatic and as much as possible the focus was on “win-win” narratives (pointing out to the gains for each group involved, rather than the potential losses). This may only be effective, however, when the change team keeps its eye on the reform goals, such that pragmatism does not lead to drift.

### Aligning technical elements of the reform

The technical content of the reform is a key element in its political economy, of course – having implications for who might gain or lose (and so support or oppose) as well as how challenging it may be to implement and to institutionalise. In these case studies, the core reforms related to pooling, which directly affects the public and so can be more politically controversial (but arguably, once enacted, harder to reverse as that would involve withdrawing benefits from often substantial population groups) [76]. The increase in risk pooling was more extensive in Thailand and Indonesia – in the case of Nepal, the stepwise extension of the maternity protection and later entitlements to free basic health care were more incremental and were undermined, especially for free basic health care, by implementation challenges. It is notable however that the policies have been maintained, even during later phases when social health insurance came into the spotlight. In Thailand and Indonesia, purchasing and provider payment reforms were also significant and undertaken strategically. For example, the choice of capitation as the main provider payment mechanism was essential in an early phase as it is often difficult to bring this in when providers are used to fee-for-service payments.

Ensuring that the health system is ready for coverage expansion is also critical. The free care policies in Nepal face ongoing challenges relating to the capacity of the health system as a whole (related to resource challenges but also political instability and fragmented policymaking). By contrast, in Thailand, the importance of earlier investments in health system strengthening are highlighted by the case study. The 2002 creation of the UCS and UHC reforms built on a “solid platform” of health system development achievements which were obtained over time, going beyond the two decades we have focused on above and also beyond health financing reforms. Key health financing reforms started in the 1970s and continued during the 1980s and 1990s^2^. Although these are considered piecemeal and fragmented and did not succeed in achieving universal coverage [27], they were critical to build on for the creation of the UCS, but also for the experience and knowledge the different actors had acquired in terms of financial protection. Similarly, the UCS scheme built on earlier health system reforms, including of public health infrastructure and human resources policies to ensure quality and availability (including in rural areas) of health workers and services [52]. In Indonesia, supply side factors such as distribution of facilities and staff have been one of the major constraints to the JKN implementation, and decentralisation has created implementation challenges in Indonesia as in Nepal.

The financing capacity of a country also interacts with technical and political components. In Nepal, which was the only low-income country in this group, not all of the reform initiatives were backed up with the required resources and commitment for effective implementation. Maternity care covered by the Aama programme was supported with the required resources, in contrast to free health care and health insurance, which have been facing limited additional resources and implementation challenges (such as stock out of tracer medicines in free health care and high drop outs in insurance). In such settings, where there is a large component of external financing to the sector, it is likely that lack of strong opposition from the MoF to planned reforms – or at least MoF not ensuring that reforms are tailored to the available resources – is linked to expectations of external support for plans, as well as an acceptance of partially implemented policies in some areas, which softens contestation.

## Conclusion

In this paper, we add to the relatively limited literature on the political economy of health financing reforms in specific settings [77], analysing not only stakeholder positions and influence in three south-east Asian countries but the role of change teams, the strategies they adopted to move reforms forward and how prior shocks and crises may have created opportunities for or enabled reforms. In a world which is acknowledged to be facing polycrises, this topic is of current policy interest. Crises can and do affect the three ‘streams’ of politics, problem and policies [4], however taking advantage of this requires leadership around the technical and political responses, which we investigate here. We do however need to acknowledge some limitations of the study, which is based on three rapid case studies from varying contexts, making comparisons challenging.

The case studies show that the broader context had a significant impact on the strategies adopted, as well as on the content of the reform. In Thailand and Indonesia, the introduction of the universal schemes took place against the backdrop of a major cultural and political shift and a move towards democracy and participation, which allowed the widespread mobilisation around UHC. At the same time, the economic crisis did not affect the reformist climate and in fact activated demands for change and a sense of urgency to achieve this change in a rapid manner, becoming in fact a catalyst for reform. This is not dissimilar from post-war Britain, when the NHS was created against the backdrop of a very challenging economic situation [78], or health system reforms after the Russian invasion of 2014 in Ukraine [79]. In Nepal, while the successful peace building process triggered the provision of free health services as part of a ‘peace dividend’ and new populist politics, the prolonged political instability since then, the low availability of domestic financial resources and the introduction of major structural reforms such as federalisation have adversely affected the implementation of the reforms and their effectiveness.

Looking across the three case studies (Table 9) and comparing with the list of possible strategies in the WHO PEA guide [3], we see that many strategies were deployed, with the largest number being in Thailand but all using a good range. This demonstrates the importance of strong technical teams that can directly engage with and incorporate political economy dynamics into reform strategies. In doing so, they can develop reform strategies that either address or make strategic compromises to ensure reforms, and the objectives they support, continue to make progress. These findings align with those of previous studies that have examined how reform teams proactively manage political economy dynamics in moving health financing reforms forward [77]. This includes the influential role of a well-informed executive; the importance of mobilizing and organizing public support behind a reform to enable resilience; and strategic engagement with external actors and research institutions to generate evidence bases to inform both technical and political strategies. They also highlight critical points of opposition, including inter-bureaucratic resistance and medical associations, where strategies can be employed to sway or divide parts of stakeholder groups that dissipates their power and influence in a reform process. Elements in the Nepal case also highlight that lack of strong resistance by budget holding stakeholders can occur when no real shift to resource allocation is expected (when it is expected that policies will be externally financed or under-financed, for example).

Reform management strategies must of course reflect varying contexts and goals, but the case studies do highlight some preliminary insights, such as: the value of a change team that is well-connected, clear in its goals and technically as well as politically savvy; the importance of connecting to social values to entrench reforms; the value of changing decision rules to reduce the veto power of opponents (now and in the future); the important role of participation and transparency in mobilising the support of beneficiaries and civil society; the strategic use of dialogue to create win-win situations; the benefits of speed when more organised opposition is anticipated; and the need for iteration and flexibility in reforms, while keeping a clear goal in mind. Importantly, these case studies highlight the art of the possible in terms of moving health financing reforms forward that advance UHC objectives. They underscore that political economy analysis can provide more than understanding of context, and can be an integral part of determining the content, design, and implementation of reforms.

Most PEA studies have adopted retrospective analysis [80] and further work is needed to guide on the prospective use of PEA tools to support effective reformers. Too many people continue to have inadequate financial protection and access to quality health care. As we look ahead to inevitable future crises and shocks, PEA can be one component of a flexible toolbox of analytics to prepare for and navigate windows of opportunity to advance UHC. The technical specifications of reform processes are often well-documented, without sufficient attention to building the evidence base on country-level examples of PEA strategies and their outcomes for health financing and other reforms.

## Electronic supplementary material

Below is the link to the electronic supplementary material.

Supplementary Material 1

## Data Availability

Data is provided within the manuscript, its references or supplementary information files.

## Footnotes

1 Measured as catastrophic out of pocket health spending at 10% of threshold.

2 In the previous decades, other key health financing reforms were introduced which created a solid base for the latter reforms. These include the Scheme for low-income and vulnerable populations (1975), CSMBS for the government employees (1980), Voluntary health insurance for the informal sector (1983), Social Health Insurance for private sector employees (1990) [52].

## Abbreviations

AFC: Asian financial crisis
ART: Anti-retroviral therapy
BHSP: Basic health services package
CHE: Current health expenditure
CPA: Comprehensive peace agreement
CPN-UML: Communist party of Nepal-united marxist leninist
CSMBS: Civil servant medical benefit scheme
DRG: Diagnostic related groups
EDPs: External development partners
GDP: Gross domestic product
GNI: Gross national income
ILO: International labour organisation
IMF: International monetary fund
INA-CBGs: Indonesian case-based groups
JKN: Jaminan kesehatan nasional
KAJS: *Komite Aksi Jaminan Sosial*/Social security action committee
KIIs: Key informant interviews
LDC: Least developed country
MOF: Ministry of finance
MOH: Ministry of health
MOHP: Ministry of health and population
MOPH: Ministry of public health
MWS: Medical welfare scheme
NCDs: Non-communicable diseases
NGOs: Non-governmental organizations
NHSB: National health security board
NHSO: National health security office
NSSA: National social security act
OOP: Out-of-pocket
PEA: Political economy analysis
Puskesmas: Public primary health care
SDIP: Safe delivery incentive programme
SEA: South-east Asia
SEAR: South-east Asia region
SHI: Social health insurance
SSIA: Social security implementing agency
SSS: Social security scheme
SWAp: Sector wide approach
TRT: Thai rak thai
UCS: Universal coverage scheme
UHC: Universal health coverage
UUD: *Undang-undang dasar*
WHO: World health organisation
