# Supplementary material for: Political economy analysis of health financing reforms in times of crisis: findings from three case studies in south-east Asia
Source: Int J Equity Health. 2025 Feb 3;24:34. doi: 10.1186/s12939-025-02395-5 (PMC11792183; doi:10.1186/s12939-025-02395-5)
Supplement: Supplementary file 1 — Supplementary Material 1 [file 12939_2025_2395_MOESM1_ESM.docx]

**Political economy analysis of health financing reforms in times of crisis: south-east Asia case studies**

**Topic guide for key informant interviews^[[1]](#footnote-1)^**

| **Domain** | **Topic** | **Key questions** |
| --- | --- | --- |
| **Introduction** | Respondent | Could you please describe to me your role and primary responsibilities in relation with the health financing reform?  Which elements/phases of the reform process are you most familiar with? [probe: for example, agenda setting, design, adoption, implementation – can be more than one] |
| **Context** | Historical legacies and crisis/shock | [*present a brief overview of documentary review findings in relation to identified shock and health financing reform(s), perhaps using a timeline if helpful, and refer to those throughout the interview*]  What is the past history of the health financing sub-sector, including previous reforms/changes and experience of crisis?  How did the [specific crisis / provide detail] affected health financing reform(s)?  Why was it a turning point / how did it provide a window of opportunity for reform? (for example, in terms how the crisis changed the political economy dynamics around health financing reforms and UHC) |
|  | Ideologies and values | What are the dominant ideologies and value, which shape views around health financing reforms? To what extent were these influenced by the shock/crisis [mention the specific shock]? |
| **Actors & distribution of resources** | Roles and power relationships | Who are the key stakeholders involved in the decision-making process? [probe using categories and examples in framework/Figure 2]  What are their formal/informal roles and mandates in relation to the health financing reform?  To what extent was power vested in the hands of specific individuals or groups in relation the health financing reform? |
|  | Support to health financing reform | Which groups or individuals supported the health financing reform, which opposed it and which remained neutral?  What were the stakes around the health financing reform? Who were the “winners” and “losers” from it? What were the main views and who are the key supporters of it? Why?  How much political priority did the health financing reform have and why? |
|  | Ownership structure and financing | How are changes in health financing financed? What were the consequences of reform on financing structures? |
|  | Actors & context | How have features of the context, in particular those in relation to crisis/shocks/shifts [probe using relevant example(s)], have changed the actors involved and/or their roles and power relations? |
| **Process** | Decision making (agenda setting, design and adoption) | How was the reform process (at agenda setting, design, adoption and implementation stages) managed by key stakeholders?  How were decisions on the health financing reform made? Who was party to these decision-making processes? Which formal or informal forums or processes were used for consensus building and decision-making?  What role did evidence play? |
|  | Strategies to manage trade-offs and ensure progress reform | How were trade-offs managed?  How do different interest groups outside government (e.g. private sector, external organisations, non-governmental organisations, consumer groups, the media) seek to influence policy?  What strategies (including negotiation, coalition building, compromises, sequencing of reforms, etc.) were adopted by key stakeholders to ensure the progress of health financing reforms?  How do these strategies specifically adapt and reflect the context and the features of the identified crisis? |
|  | Implementation | What are the consequences of the implementation of health financing reforms? Who bears the impact, financial costs, and any potential risks?  Were particular social, regional or specific groups (ethnic, disabled, displaced, vulnerable) included or excluded?  Were the views of the front-line implementers included in the reform process?  What were the practical challenges and bottlenecks to implementation? |
| **Content** | Key features | What do you think are the key features of the health financing reform that ensured it success? [probe: any technical detail in design or implementation or decision of processes that you think was particularly innovative, break with previous tradition, represented a successful outcome of lengthy negotiations, etc.) |
|  | Outcomes of strategies adopted (adaptations) | What were the outcomes of strategies adopted to manage trade-offs and ensure progress reform for example in terms of changes in content or approach in design and implementation practices?  What changes in the content of the health financing reform or in the implementation approach had persuade some of the opponents to be more supportive? Why?  Where there any adaptations or compromises in the design or implementation approach? |
|  | Crisis and policy content / adaptations | How far did the health financing reform(s) respond to, or reflect historical legacies and dominant ideologies and values (including how they shifted during crisis)?  Did any of the adaptations or compromises mentioned above in design and/or implementation approach related specifically to the crisis, and if so how? |

1. Adapted from

   DfID (2009), Political economy analysis: how to note. London: Department for International Development. <https://www.odi.org/sites/odi.org.uk/files/odi-assets/events-documents/3797.pdf>

   Witter et al (2019), The political economy of results-based financing: the experience of the health sys em in Zimbabwe. Global Health Research and Policy, 4:20. <https://ghrp.biomedcentral.com/articles/10.1186/s41256-019-0111-5>

   Reich M, Campos P (2020), A Guide to Applied Political Analysis for Health Reform. [↑](#footnote-ref-1)
